# Measuring the Impact of Data Sharing: From Author-Level Metrics to Quantification of Economic and Non-tangible Benefits

**DOI:** 10.7759/cureus.50308

**Published:** 2023-12-11

**Authors:** Enzo Emanuele, Piercarlo Minoretti

**Affiliations:** 1 Occupational Health, 2E Science, Robbio, ITA; 2 Occupational Health, Studio Minoretti, Oggiono, ITA

**Keywords:** open data, cost savings, evaluation metrics, impact, data sharing

## Abstract

In early 2023, the National Institutes of Health (NIH) implemented its Data Management and Sharing (DMS) Policy, requiring researchers to share scientific data produced with NIH funding. The policy's objective is to amplify the benefits of public investment in research by promoting the dissemination and reusability of primary data. Given this backdrop, identifying a robust methodology to assess the impact of data sharing across diverse research domains is essential. In this review, we adopted two methodological paradigms, the bottom-up and top-down strategies, and employed content analysis to pinpoint established methodologies and innovative practices within this intricate field. Although numerous author-level metrics are available to gauge the impact of data sharing, their application is still limited. Non-traditional metrics, encompassing economic (e.g., cost savings) and intangible benefits, presently appear to hold more potential for evaluating the impact of primary data sharing. Finally, we address the primary obstacles encountered by open data policies and introduce an innovative "Shared model for shared data" framework to bolster data sharing practices and refine evaluation metrics.

## Introduction and background

The advent of the digital age has brought about a paradigm shift in the way scientific data can be disseminated, shared, and reused [1,2]. The National Institutes of Health (NIH) has recently enforced its Data Management and Sharing (DMS) Policy, which mandates researchers to share data generated with NIH funding [3]. This policy is aimed at enhancing the dissemination and reuse of scientific data, thereby maximizing the impact of public investment in research [4]. The implementation of this policy has underscored the necessity for a dependable methodology to assess the impact of data sharing across diverse research fields. Despite the potential advantages of sharing practices, there is currently no widespread agreement on how to effectively measure the impact of open data [5-7].

This review aims to assess potential strategies for evaluating the influence of data sharing at different levels, employing two methodological paradigms, namely, the bottom-up and top-down strategies. Content analysis was then utilized to identify established methodologies and innovative practices in the field. We also briefly address current barriers faced by open data policies and propose a novel "Shared model for shared data" framework to enhance data sharing practices and improve evaluation metrics. Our overarching goal is to provide a comprehensive understanding of the current landscape of data sharing impact evaluation and propose innovative strategies for future research in this area.

## Review

Methods

Search Strategy and Methodological Approach

To evaluate the significance of data sharing, particularly in relation to primary data, our literature review employed two methodological paradigms: the bottom-up and top-down approaches [8]. By employing these strategies, we were able to gain a holistic understanding of the subject matter, enabling us to recognize established methodologies as well as novel practices within the field. The bottom-up methodology started at a granular level, examining specific studies or instances, and incrementally moved toward a more encompassing understanding [8]. For the purpose of data sharing, our initial step was to scrutinize individual case studies or research articles that tackled data sharing within specific biomedical research areas such as genomics, proteomics, or neuroimaging. This process entailed an in-depth analysis of how data sharing was operationalized in these particular instances, the obstacles encountered, the advantages gained, and the quantifiable impact of data sharing on the results. This methodical approach was instrumental in progressively constructing a holistic understanding of data sharing's influence across the entirety of biomedical research.

Considering that the DMS Policy primarily focuses on facilitating the distribution of scientific data and speeding up progress in biomedical research [3,4], the selected methodology initially involved an exploration of PubMed. The research approach for investigating the impact of data sharing was initially defined using the following search criteria in PubMed: (("data"[Title/Abstract] OR "sequence data"[Title/Abstract] OR "software"[Title/Abstract] OR "dataset"[Title/Abstract]) AND ("sharing"[Title/Abstract] OR "impact"[Title/Abstract])) AND ("evaluation"[Title/Abstract] OR "assessment"[Title/Abstract] OR "effectiveness"[Title/Abstract] OR "outcomes"[Title/Abstract] OR "qualitative"[Title/Abstract] OR "quantitative"[Title/Abstract] OR "survey"[Title/Abstract]). The database search was restricted to the timeframe spanning January 1, 2013, to September 30, 2023, yielding a substantial 1,193 entries pertinent to the key research topics. To broaden the research scope, the search strategy was subsequently augmented to include a diverse array of databases such as Science Direct, Web of Science, Scopus, and Google Scholar. Our objective was to identify articles that prominently incorporated terms such as "data sharing," "data sharing policies," "open-access data," "data re-use," "data privacy," and "data security" within their titles or abstracts, in conjunction with terms such as "impact," "outcomes," and "consequences." In the pursuit of understanding the impact of data sharing in biomedical research, we subsequently employed a top-down approach, beginning with a comprehensive overview before delving into specific components [8].

We initially analyzed a collection of meta-analyses, reviews, systematic reviews, and protocols that provided a high-level perspective on data sharing's implications. This approach offered an understanding of the overarching trends, potential benefits, and challenges that accompany data sharing. Subsequently, we narrowed our focus to examine these trends in specific sub-disciplines or even single studies, supplementing the macroscopic view with a microscopic examination. Our research utilized the following search criteria in PubMed to ensure a comprehensive review: "data" AND "sharing" AND "impact"; "data" AND "sharing" AND "advantages"; "data" AND "sharing" AND "obstacles"; "data" AND "sharing" AND "trends"; "data" AND "sharing" AND "sub-disciplines." To further refine our top-down search results, only articles classified as meta-analysis, review, systematic review, and protocols were considered. The final selection of works was also sourced from reputable scientific databases, including Science Direct, Web of Science, Scopus, and Google Scholar, emphasizing projects related to the impact assessment of data sharing.

Synthesizing the Outcomes From the Literature: Content Analysis

The literature acquired through the application of both bottom-up and top-down techniques was meticulously scrutinized using content analysis [9]. This method, primarily employed in qualitative research, serves as an organizational tool that prompts potential implications from the amassed data, thereby paving the way for new discoveries. Content analysis was subsequently carried out to meticulously analyze and categorize the retrieved literature. This approach empowers researchers to probe into prior analyses, extracting additional results from empirical findings [10].

Results

A careful examination of the current body of literature unveils two distinct approaches for evaluating the influence of this practice, i.e., author-level metrics and non-conventional metrics. Each of these methods offers a unique perspective on the repercussions of data sharing. In the following sections, we will embark on a comprehensive exploration of these methodologies.

Author-Level Metrics

Despite certain concerns about equity and fairness, author-level metrics are a commonly utilized gauge of personal scientific achievement [11,12]. Quantitative tools such as the h-index and its derivatives are engineered to measure both the volume of publication output, indicated by the total count of published materials, and the influence of research, denoted by the number of citations garnered [12]. These metrics often hold a critical place in decision-making procedures, where they can profoundly affect the allocation of resources and recruitment decisions within academia [12]. There is an increasing body of evidence suggesting that the practice of data sharing can amplify these author-level metrics positively. Research studies that offer open access to their data are generally observed to attract more citations compared to those that keep their data private [13]. According to a cross-disciplinary investigation by Colavizza et al. [14], papers that indicate data availability are cited, on average, 25% more. In microarray-based research, articles providing access to raw data accumulate an average of 69% more citations in contrast to other articles [15]. Further, a separate study revealed that articles providing access to their data receive an additional 97 citations, with a standard error estimate of 34 [5]. Even though the prospect of enhanced scholarly citations presents a potential reward for authors who make their data publicly accessible, it has not been a strong enough motivation for a significant number of researchers to disclose their data [13,16]. This reluctance exists even in light of the fact that evaluators, including reviewers and funding bodies, take author-level metrics into account when evaluating researchers and their project proposals. However, despite these obstacles, there has been progress in the form of the development of various author-contributor systems specifically designed to measure the impact of data sharing.

S-index

The S-index, proposed by Olfson et al. [17] in 2017, is a metric designed to encourage researchers to share their data by measuring the impact of shared data on scientific publications. They define the S-index as follows: each researcher who shares their data is considered, and the publications that employ their shared data are listed in the order of citation frequency. The S-index is then determined by the highest number of papers (n) on this list that have received n or more citations [17]. Essentially, all publications citing data shared by the author are ranked, and the S-index is found by identifying the highest number of papers (n) with a minimum of n citations. The authors note that the S-index aligns with the suggestion of distinguishing "data authors" from regular authors [18]. Ascoli [19] and Ascoli et al. [20] proposed that the S-index could motivate researchers to share data and code, and it could function independently of existing publication indices. Conversely, Kattge et al. [21] have suggested that there should be no differentiation between data citations and publication citations when creating performance metrics. The authors advised that the establishment of a distinct "data h-index" might inadvertently result in data contribution being perceived as an inferior performance metric [21]. Table 1 depicts the primary advantages and disadvantages of the S-index.

**Table 1:** Primary advantages and disadvantages of the S-index

Advantages of the S-index	Disadvantages of the S-index
It incentivizes researchers to share their data, leading to more collaboration and scientific progress.	It may not be a fair measure of a researcher's impact if they do not share their data or if their data is not widely used.
It takes into account the impact of a researcher's shared data on other publications, providing a more comprehensive measure of their influence.	It may be biased toward researchers who work in fields where data sharing is more common or necessary.
By ranking publications based on the use of shared data, the S-index acknowledges the contributions of researchers who make their data available to others.	Since 2017, it does not appear to have gained widespread adoption as a metric for evaluating researchers. As of October 1, 2023, the original methodology's description has attracted a modest 25 citations since its publication.

Data-index

The data-index, introduced by Hood and Sutherland in 2021 [22], is derived in a similar manner to the h-index, but instead of ranking publications based on their citations, it prioritizes original datasets based on their data-index citations. Essentially, an author's data-index corresponds to the quantity of datasets (n) that they have contributed to (either as a primary author or coauthor), which have garnered n or more data-index citations. It is important to distinguish between data-index citations and data citations; the former is calculated by aggregating first- and higher-level citations (i.e., citations of datasets or articles that have referenced the original dataset), whereas the latter only involves summing the first-level citations of a dataset [22]. However, only higher-level citations derived from datasets or publications that have reprocessed the original dataset are incorporated in the data-index citations. This indicates that whenever their data are cited, originators of the initial dataset accrue data-index citations, regardless of their authorship status in the datasets or publications that have repurposed their data [22]. However, it is feasible that data-index citations from the second level or higher might be attributed to datasets not reusing data from the original dataset. Table 2 depicts the primary advantages and disadvantages of the data-index.

**Table 2:** Primary advantages and disadvantages of the data-index

Advantages of the data-index	Disadvantages of the data-index
It employs citations as a benchmark to gauge the impact of shared data. This provides a quantifiable standard to measure the extent of influence exerted by open data within the scientific community.	If a dataset is not frequently cited, it may not be correctly represented in terms of its actual importance or impact in the data-index.
Datasets that have been instrumental in shaping prominent theories can be recognized and rewarded through the data-index.	It primarily values citations over frequency of reuse, which may not fully reflect the usefulness or applicability of a dataset.
It promotes transparency and reproducibility in research.	It might unintentionally favor datasets in popular or trending research fields that naturally attract more citations, potentially marginalizing less popular but equally important areas.

SCIENCE-index Augmented

Adams et al. [23] have recently taken a commendable step toward fostering a culture of data sharing and collaboration in scientific research by refining their original SCIENCE-index model. The improved version, named the SCIENCE-index Augmented, leverages the robustness of blockchain technology to provide a more comprehensive evaluation of a researcher's scientific contributions, including data sharing [23]. The SCIENCE-index Augmented is a comprehensive system that evaluates researchers' contributions using five key parameters. These include career length, paper count, citation count, data share count (squared), and h-index. The career length parameter signifies the total period of a researcher's career, reflecting their enduring contribution to their field. Paper count represents the aggregate number of papers a researcher has published, serving as a gauge of their productivity. Citation count indicates the frequency with which other researchers have referenced a particular researcher's papers, signifying the impact of their work. Data share count (squared) underscores the significance of data sharing in fostering open and collaborative scientific research. Lastly, the h-index captures both the productivity and citation impact of a researcher's work, calculated from their most cited papers and the number of citations they have received in other publications.

The updated model includes a measure of a researcher’s data sharing frequency, thus serving as tangible proof of their active involvement in collaborative research. The SCIENCE-index Augmented increased progressively as a researcher increasingly partakes in data sharing, owing to its specifically designed computation formula [23]. Table 3 shows the primary advantages and disadvantages of the SCIENCE-index Augmented.

**Table 3:** Primary advantages and disadvantages of the SCIENCE-index Augmented

Advantages of the SCIENCE-index Augmented	Disadvantages of the SCIENCE-index Augmented
It can be used and weighted to augment the h-index in favor of data sharing.	Its survival and scalability depend on researchers being properly incentivized to participate.
It can predict the future progress of a researcher based on their past contributions to science.	The blockchain-based model must be computationally lightweight to avoid high transaction costs when computing on a public virtual machine yet robust enough to rate researchers accurately.
It includes data sharing statistics as a parameter, rewarding researchers for their data sharing efforts and incentivizing further data sharing.	It may introduce flaws as it might be easier to publish data than a paper, which could lead to artificially inflating a researcher's SCIENCE-index Augmented when less valuable datasets are shared.

It is worth noting that the three identified author-level metrics, which are intended to measure the impact of data sharing, have not achieved the same level of acceptance as the traditional h-index [24]. This disparity can be attributed to a few factors. Firstly, the h-index has a longer history and wider recognition. Secondly, the h-index is comparatively straightforward to calculate and comprehend, while some of the data sharing metrics may be more intricate or demanding to compute. Lastly, the h-index generally serves as a comprehensive measure of research impact, whereas data sharing metrics target a specific research aspect. Nevertheless, as the scientific community places increasing value on data sharing, it is plausible that these metrics will gain broader acceptance and usage.

Non-conventional Metrics: Economic and Non-tangible Benefits

Non-conventional metrics have also been proposed to assess the impact of primary data sharing. Among them, economic (i.e., cost savings) and non-tangible benefits are promising and will be examined.

Economic Benefits

Data sharing may serve as a cost-effective measure for researchers, bypassing the financial burden associated with fresh data generation for each individual study [25]. This concept is aptly demonstrated in the case study by Milham et al. [26], which utilized magnetic resonance imaging (MRI) data collected and distributed through the International Neuroimaging Data-sharing Initiative (INDI). INDI is a synergistic initiative in the realm of neuroimaging, championing the principles of Open Science through the sharing and accessibility of extensive brain imaging datasets [26]. This project broadens the scope for researchers to tap into a diverse range of neuroimaging data, thereby enriching our comprehension of the human brain. The INDI project houses several specific datasets, including the Functional Connectivity Project (FCP), the Nathan Kline Institute-Rockland Sample (NKI-RS), the Attention Deficit Hyperactivity Disorder-200 (ADHD-200), the Autism Brain Imaging Data Exchange (ABIDE), and the Connectome Computation Research Repository (CoRR) [26]. These datasets comprise neuroimaging data procured from individuals suffering from various neurological and psychiatric disorders, such as ADHD and autism. With the understanding that the cost of MRI studies can fluctuate significantly based on the targeted population, Milham et al. [26] projected the costs associated with recruitment, phenotyping, and imaging in each dataset as follows: FCP: $1,000, ADHD-200: from $2,000 to $5,000, NKI-RS: $3,000, ABIDE: from $5,000 to $10,000, and CoRR: $2,000. As of 2018, each of these datasets has resulted in 308, 210, 188, 190, and 17 publications, respectively. The total sum saved by opting for data sharing over de novo data generation for each paper ranged from approximately $893,258,000 (on the conservative end) to $1,706,803,000 (on the liberal end) [26]. This case study reveals that data sharing holds significant financial merit for funding agencies. By eliminating the necessity for redundant studies, it optimizes the return on investment, thereby making it a cost-effective strategy [3].

Non-tangible Benefits

In the referenced case study, Milham et al. [26] explored the non-tangible benefits of the INDI, specifically analyzing its influence on the intellectual perspectives of authors beyond the mere provision of datasets. They discovered a total of 639 publications that acknowledged the INDI without directly utilizing the data. Additionally, they noted 71 publications that employed either the data analysis scripts from the initial FCP release manuscript or their respective derivative platforms, underscoring the initiative's utility beyond data provision [26]. Taken together, these insights underscore how INDI has promoted resource sharing and innovation, thereby cultivating a collaborative scientific community.

Discussion

Author-Level Metrics: State of the Art

Author-level metrics have not been widely adopted for evaluating the impact of data sharing. As of November 18, 2023, the S-index, introduced in 2017 [17] has seen limited acceptance, with the original methodology calculation garnering only 25 citations since its inception. The data-index methodology, established by Hood and Sutherland in 2021 [22], has likewise attracted a mere 14 citations by the same date. Significantly, these metrics are not readily available on widely used platforms such as Scopus, Google Scholar, or ResearchGate, where researchers typically monitor their h-index. In 2023, a new author-specific metric, the SCIENCE-index Augmented, was proposed by Adams et al. [23]. This metric holds considerable promise for forecasting a researcher's future trajectory based on their prior scientific contributions. Nevertheless, the computational demands of the blockchain-based model need to be minimized to prevent excessive transaction costs on public virtual machines [23]. There is also a concern that this model could facilitate simpler data publishing than paper publications, potentially causing artificial inflation of a researcher's SCIENCE-index Augmented score when less valuable datasets are shared [23].

Non-conventional Metrics: State of the Art

The necessity for robust economic evaluations concerning primary data sharing is pivotal in implementing Open Science policies [27]. To our knowledge, no existing study has yet comprehensively examined the repercussions of data sharing with formal economic analyses. However, by considering the potential for cost savings as a novel metric to discern the impact of data sharing, we can draw insights from the INDI neuroimaging project and extrapolate this concept to diverse disciplines [26]. To this aim, ophthalmology will be used as an illustrative example. In this discipline, data collection on retinal tissue biometrics employs high-cost methods such as optical coherence tomography, color retinal photography, and fluorescein angiography [28]. However, the monetary implications of these techniques can fluctuate significantly, influenced by factors such as equipment, staffing, and patient demographics. To gauge the financial impact of data sharing within ophthalmology, we can emulate the approach used by Milham et al. [26] for neuroimaging data. The first step involves focusing on a dataset that includes retinal tissue biometric data, followed by cost estimation per patient for each ophthalmic imaging technique. Subsequently, we must ascertain the number of studies that have benefitted from data sharing. Once these steps are completed, we can gauge total cost savings by juxtaposing the expenses of original data generation against the costs of data sharing for each study. Notably, this enhancement in economic evaluations of data sharing in ophthalmology could be instrumental in promoting diversity and addressing disparities among contributors from low- and middle-income countries (LMICs) [29]. Indeed, this approach could significantly aid in the sharing of invaluable data with researchers from LMICs who may not have the requisite resources for original, expensive retinal tissue biometric data collection [29]. By offering these researchers' access to such data, we can incorporate their unique insights and innovative queries, thereby enriching the broader research landscape.

Open Issues in the Field of Open Data

The intricacies of the primary data ecosystem, involving a multitude of stakeholders, make it a challenging realm. Despite the establishment of FAIR principles, which advocate for data to be Findable, Accessible, Interoperable, and Reusable [30], their adoption for data stewardship planning and application remains sparse. Consequently, a significant portion of research data across various disciplines is not currently classified as "FAIR." The concept of "dark data" has been proposed for unshared data, implying that such data, if unveiled, could potentially lead to new discoveries [31]. However, despite the broadly acknowledged benefits of data sharing, several researchers and scientists harbor a reluctance to share their data. This reluctance can be traced back to an array of factors: firstly, concerns surrounding privacy, data ownership, and intellectual property rights [32,33]; secondly, the dearth of time, funds, resources, and incentives for accomplishing the tedious task of data organization [34]; thirdly, worries over the possibility of errors embedded within the data and the quality of statistical reporting [35]; fourthly, apprehensions about the existence of sensitive information and data misuse (e.g., data leakage to health insurance companies) [36,37]; fifthly, the absence of reward or recognition for sharing data [38]; and lastly, the lack of a universal framework that enforces voluntary or mandatory data sharing [39]. Moreover, the decision to share or withhold data is not exclusively a personal choice for scientists or researchers. It is also significantly influenced by institutional and national factors that can impose restrictions on data sharing. For example, copyrighted data sources may prohibit the publication of certain types of data, creating substantial barriers in the data sharing field due to concerns about data availability rights [4]. In scenarios where there are no mandates, researchers have often exhibited a willingness to deposit some, but not all, of their data into unrestricted public data repositories [38]. This willingness increases when privacy and ethical considerations are taken into account, as well as when conditions on governance and regulation of access are imposed [38]. This underscores the critical role of appropriate policies and governance mechanisms in facilitating data sharing among scientists and researchers.

Toward a "Shared Model for Shared Data" Framework

The intricacies of assessing the impact of data sharing stem largely from the non-uniform, diverse, and sub-optimal practices prevalent in the field [40,41]. For instance, while the tradition of sharing data via supplementary tables in academic journals persists, it presents several challenges. These include the absence of peer review, less rigorous curation compared to the main article, and potential link degradation. Additionally, embedding data within journal articles does not serve as an ideal solution, as it neither assures long-term data preservation nor does it facilitate dataset-specific searches [42]. To elevate the efficacy of data sharing practices, we believe that a "Shared model for shared data" framework could prove instrumental. This system could empower stakeholders to standardize, streamline, assess, and enhance both the efficiency and effectiveness of data sharing. This framework aims to promote seamless, efficient, and effective data sharing by establishing shared standards, practices, and metrics. It seeks to create an environment where data are not only shared but also used and evaluated in a way that creates maximum value for all stakeholders. A simplified workflow toward the "Shared model for shared data" framework can be schematically divided into three phases: pre-sharing, sharing, and post-sharing.

"Shared Model for Shared Data" Framework: Pre-sharing Phase

In the "Shared model for shared data" framework, the pre-sharing phase is the crucial first step toward ensuring the quality and usability of shared data. This phase encompasses a series of steps that researchers must undertake before their data can be made available to the scientific community. The first step in the pre-sharing phase is data identification and selection. Researchers must carefully analyze and decide which collected data will be shared. This process may require substantial analysis and decision-making by researchers who are willing to share their primary data, as it involves determining which datasets will provide the most value to the scientific community when shared. Once the data is selected, the next step is data processing and cleaning. This process includes removing any errors, inconsistencies, or redundancies in the data to ensure that they are accurate and reliable. Another important step in the pre-sharing phase is data standardization and harmonization. To promote uniformity and consistency, researchers must standardize and harmonize the data. This involves applying protocols and standards such as templates, formats, terminologies, and taxonomies. Ideally, a common data model (CDM) [43] should be established during this phase, which provides an efficient approach to organizing and structuring the data. Standardization and harmonization not only make the data easier to understand and analyze but also enhance compatibility across different systems and platforms. In addition, data protection and compliance are essential considerations during the pre-sharing phase. Researchers must ensure that the data they plan to share comply with relevant regulations and standards, such as data protection, anonymization, and privacy laws [36,37]. This step is crucial to safeguarding the privacy and confidentiality of individuals involved in the data. Clear terms of data use permission should also be established before sharing the data. Researchers need to define how different types of data can be shared, with whom, at what level, and under what circumstances [44]. It is important for researchers to have a clear understanding of data ownership and the rights that data owners possess.

Once all these steps are completed, researchers can proceed to the data submission stage. Similar to submitting academic papers, the data is submitted to a repository or platform that is accessible to others. Submission of primary data for sharing should be followed by peer review. Similar to the peer review process for academic papers, implementing a peer review system for data during the pre-sharing phase ensures the quality and reliability of the data [45]. This rigorous evaluation not only validates the data but also enhances the credibility and importance of data sharing as an integral part of scientific research. A study conducted by Roche et al. [46] analyzed 362 open datasets published by 100 principal investigators in the fields of ecology and evolution over a span of seven years. The findings revealed poor data quality scores, with only 56.4% being deemed complete and 45.9% being reusable [46]. This highlights the insufficiency of relying solely on open data policies to guarantee data quality and emphasizes the necessity of pre-sharing peer review to ensure completeness and reusability. Pre-sharing peer review plays a vital role in the "Shared model for shared data" framework.

"Shared Model for Shared Data" Framework: Sharing Phase

Upon successful peer review, the data are approved for publication in a data sharing repository, thus commencing the sharing phase. In addition to institutional and journal-supported repositories, there is an urgent need for the development of disciplinary repositories. These specialized data storage systems are crucial to enhancing data sharing practices within specific fields of study. This will not only improve accessibility but also foster collaboration, thereby driving advancements in each respective discipline. The sharing phase signifies the juncture at which the data are distributed among diverse stakeholders. The first step in the pre-sharing phase is metadata annotation. The mere dissemination of primary data frequently falls short of enabling efficacious reuse. To truly tap into their potential, it is essential that these datasets are complemented with extensive metadata, which illuminate their context, elucidate their source, and explain their configuration [47]. Metadata also serve a crucial role in providing pivotal specifics such as the identity of the data's originator, the timestamp of its creation, its structural format, and any corresponding keywords, thereby enriching the data's overall comprehensibility.

The second step is to assign a Digital Object Identifier (DOI) to the shared data. A DOI is a unique alphanumeric string that provides a persistent link to the data and helps in its identification and citation [48]. Registering a DOI for the shared data ensures its long-term accessibility and citability. Once a DOI is assigned, the peer-reviewed shared data can be published. Publishing involves making the data available to the intended audience through a suitable platform or repository. This step ensures that the shared data is discoverable and accessible to the stakeholders. In some cases, it may be necessary to export the shared data in eXtensible Markup Language (XML) format. XML is a widely used format for structuring and encoding data. Exporting the data in XML format ensures its compatibility with various systems and facilitates its integration and reuse. After the data are published in the desired format, they can be distributed externally to the intended users or organizations. This can be done through various means, such as sharing the data files via secure file transfer protocols or providing access to a shared data repository. Alongside external distribution, it is important to maintain an internal archive of the shared data. This ensures that a backup copy of the data is retained within the organization for future reference and retrieval. Internal archiving also helps in maintaining data integrity and security.

The subsequent phase of the sharing process involves disseminating information about the shared data to the relevant stakeholders. This can include notifying potential users or organizations about the availability of the data, highlighting its features and potential applications, and encouraging its utilization for research, analysis, or other purposes. When sharing data, it is crucial to ensure security and privacy [36,37]. This involves putting measures in place to protect the data from unauthorized access or breaches. This should also comply with any relevant data protection laws and regulations. Finally, data governance policies must be put in place. This involves the management of data assets across the organization or between organizations. It ensures that there are clear policies and processes in place for data sharing, including who can access the data, how they can use it, and how the data is maintained and updated.

"Shared Model for Shared Data" Framework: Post-sharing Phase

Upon the publication of data, it is crucial to implement a range of measures to ensure the efficient utilization and governance of the shared data. Additionally, it is paramount to evaluate the impact of shared data at various levels, including the economic aspect. The first step in the post-sharing phase is to establish clear data access and usage policy, which must unambiguously delineate the entities possessing the rights to access shared data, along with the specific scenarios that warrant such access. The policy may incorporate a role-based access control system, which offers diverse user groups differing degrees of data access, contingent on their individual roles. Concurrently, it is crucial to devise and put into action a comprehensive usage policy that ensures the ethical utilization of shared data [49]. It is advisable to shun ad hoc negotiations and data acquisitions. Moreover, monitoring and auditing mechanisms should be in place to ensure adherence to these policies.

The second step is to foster a culture of data recognition and ensure that authors receive adequate credit for their work. It is essential to establish standardized data citation-based metrics across disciplines, journals, and data repositories. These metrics should be transparent, replicable, and universally applicable to ensure consistency and fairness. To further promote transparency and accountability in data usage, implementing Counting Online Usage of NeTworked Electronic Resources (COUNTER) compliant usage statistics [50] is recommended. This will provide clear, comparable, and reliable data on the usage of shared data, facilitating the identification of usage trends and potentially highlighting areas of interest or concern. Regular collection and analysis of data reuse metrics can provide valuable insights into the impact and reach of shared data. By comparing these metrics with usage statistics, one can identify correlations and trends, informing future data sharing indicators and policies. Despite the increasing recognition of the value of data sharing and open research practices, there are currently insufficient reward mechanisms to motivate such behavior [51]. The Open Scienceframework has introduced badges that can be assigned to journal articles adhering to Open Science practices [52], but uptake is currently limited. To address this, the research community could consider developing more robust reward systems, such as formal recognition of data sharing in academic evaluations and promotions, or financial incentives. Notably, the "Shared model for shared data" framework will enable the pooling of data from diverse sources. This approach not only increases sample sizes but also addresses bias, making it instrumental in driving robust scientific progress. However, it is imperative to emphasize the importance of recognizing and appreciating the inputs of the original data contributors. This can be achieved through appropriate incentives. Acknowledging their valuable input not only fosters respect but also validates the integrity of the merged datasets. In addition, it will become imperative to track the long-term financial implications of shared datasets for funding bodies. These shared resources have the potential to eradicate the need for repetitive studies, thereby enhancing monetary efficiency. Furthermore, proactive measures need to be implemented to encourage data sharing with contributors from LMICs [29]. These researchers often face financial hurdles in generating primary data, and their inclusion in the data-sharing ecosystem can foster diversity and address scientific disparities across nations.

## Conclusions

The current landscape of data sharing exhibits a growing disparity between escalating public support for data sharing and the limited acceptance and diffusion of quantitative metrics designed to assess its actual impact. Despite universal recognition of data sharing's advantages, a pervasive reluctance to share data persists, largely due to the absence of a universally accepted institutional framework. We believe that there are valid reasons for this hesitancy in the absence of shared regulations. Additionally, the implementation of author-level metrics to quantify individual contributions to data sharing poses significant challenges and does not encourage this practice in terms of individual recognition. In this paper, our argument centers around the compelling economic benefits that can be derived from sharing expensive-to-generate data, such as neuroimaging or "multi-omic" biomarker data. By emphasizing these benefits, we believe that institutions can gather the necessary momentum to prioritize primary data sharing. Importantly, this strategic approach holds significant potential for promoting diversity and addressing disparities among contributors from LMICs. To truly enhance inclusivity, it is crucial to acknowledge and overcome the physical, social, and regulatory challenges faced by these nations. One of the key obstacles, for example, is the limited internet access and slow connections experienced by LMICs, which hinder their active participation in data sharing initiatives. However, it is important to recognize that these countries may possess in situ data sharing mechanisms that can be harnessed to overcome some of these challenges. It is imperative to conduct additional studies to delve into the possibilities and constraints of data sharing in these particular contexts. In essence, our proposed framework, the "Shared model for shared data," offers a comprehensive structure that proactively addresses the primary obstacles currently impeding data sharing. In addition, by specifically addressing disparities and harnessing the existing data sharing mechanisms in LMICs, we can establish a more inclusive and efficient data ecosystem.
